# Milking time behavior of dairy cows in a free-flow automated milking system

**DOI:** 10.3168/jdsc.2022-0243

**Published:** 2022-07-14

**Authors:** Laura Solano, Courtney Halbach, Thomas B. Bennett, Nigel B. Cook

**Affiliations:** 1 Lactanet, Canada, Sainte-Anne-de-Bellevue, Québec H9X 3R4, Canada; 2 School of Veterinary Medicine, University of Wisconsin–Madison, Madison 53706

## Abstract

The objective of this preliminary observational study was to determine milking time behavior of cows in a free-flow automated (robotic) milking system (AMS) and identify potential factors that influenced the time waiting to be milked. Milking time behavior of 40 cows from 1 pen on a commercial dairy farm with a free-flow AMS was evaluated using video analysis over 2 d. For each study cow, data were assessed for waiting time to access the milking robot, the use of the fetch pen, robot refusals, and their lying behavior. On average, cows visited the robot to wait to be milked 6 times per day, for 15 min per visit, for a total daily waiting time of 88 min per cow (range 5 to 322 min). Daily waiting time was longer for primiparous cows and decreased with increasing days in milk, but this effect interacted with parity. Daily waiting time and number of visits to the robot were associated with voluntary use of the fetch pen. Furthermore, cows with long daily waiting times had shorter daily lying times compared with cows with short daily waiting times (9.5 vs. 11.1 h/d). It is possible that factors related to the design and layout of the AMS entry and fetch pen had an effect on waiting behavior. We inferred that adoption of grouping strategies intended to reduce competitive behavior, especially toward primiparous cows, could improve milking time behavior in a free-flow AMS. This preliminary observational data from a single herd highlights the need to confirm the findings across multiple AMS herds, both with free-flow and guided-flow systems.

## Highlights

• On average, cows waited to access the robot 1.5 hours/day, ranging from 5 minutes/day to 5 hours/day.
• Waiting behavior variability was attributed to parity, days in milk, fetch pen use, and refusals.
• Robot area entry and exit design possibly influenced competitive and waiting behavior.
• Longer waiting time was associated with shorter lying times.
• Adopting management strategies and design features intended to reduce competition may improve waiting behavior.

## Graphical Abstract

**Figure:**
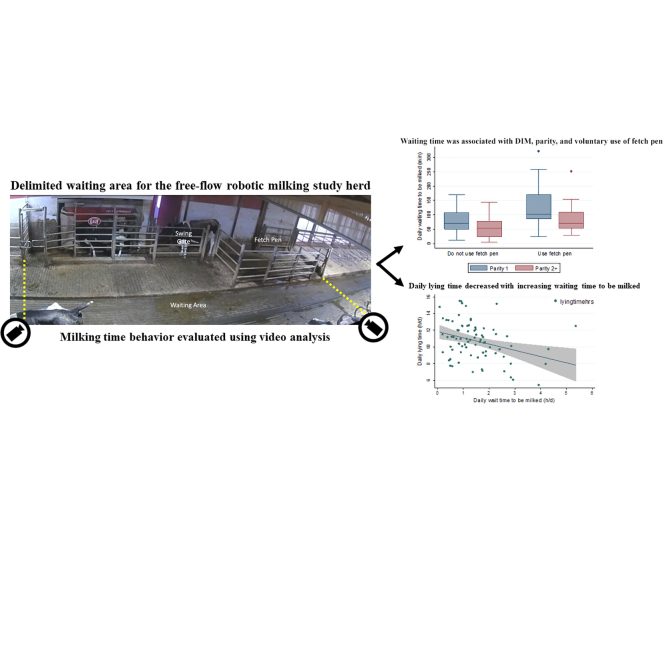
**Summary:** It is assumed that cows spend little time waiting for milking in free-flow automated milking systems (AMS), but this has not been assessed. We evaluated cows' (n = 40) milking time behavior and identified factors that influenced the time waiting to be milked in a free-flow AMS herd using video analysis over 2 days. Visit frequency and waiting time to access the robot to be milked were influenced by cows' stage of lactation and parity along with the robot's layout and design. In addition, longer waiting time was associated with shorter lying times. This study highlights the importance of minimizing waiting time to enhance cow comfort by reducing the risk for lameness.

One of the main behavioral advantages suggested for free-flow automated (robotic) milking systems (**AMS**) is that cows freely choose when to be milked without being removed from their home pen, where they rest and eat, to be transferred to a holding area adjacent to the parlor. In parlor-milked herds, prolonged milking and waiting times negatively affect the time available for rest (Gomez and Cook, 2010; Charlton et al., 2014), which is detrimental for cows' health, most notably increasing the probability for developing lameness and hoof lesions (Proudfoot et al., 2010; Sepúlveda-Varas et al., 2018).

Studies have identified that lying behavior in AMS is influenced by factors such as feedbunk space and feed push-ups (Deming et al., 2013). Of interest is that mean herd daily lying times in AMS were not notably different from those observed in parlor-milked herds [e.g., 10.5 to 12.0 h/d in freestalls with parlors (Ito et al., 2014; Solano et al., 2016) vs. 10.6 to 11.5 h/d in freestalls with AMS (King et al., 2016; Westin et al., 2016)], and fall short of the desired mean of 11.5 to 12.5 h/d (Cook, 2020). Indeed, recent studies suggest that lameness prevalence in AMS is the same or even higher than that observed in conventional herds [e.g., 21–25% of lameness in freestalls with parlors (Espejo and Endres, 2007; Solano et al., 2015) vs. 17–30% in freestalls with AMS (King et al., 2016; Westin et al., 2016; Salfer et al., 2018)].

Milking time behavior in AMS has not been examined in detail in commercial herds, nor has its impact on resting behavior. Robotic milking system manufacturers have focused on optimizing milking speed and box time so that each AMS can support a greater number of theoretical milkings/d and a larger group size, which would defray the cost of the unit over a larger number of cows. However, these calculations fail to consider the crepuscular nature of cows and potential differences in the desire to be milked throughout the day (Winter and Hillerton, 1995), which would affect robot group size. This behavior may result in periods during the day where cows are left to wait for prolonged time for their opportunity to access the robot, which may be further exacerbated by disruptions, such as robot maintenance along with fetching activity if that is mistimed.

Individual cow waiting time to be milked has not been examined in a commercial free-flow AMS because there is no gate to alert the producer that a cow may not be able to enter the robot when she wishes. Mixed-parity groups are commonplace in AMS design and data are scarce to inform us of the impact of parity on waiting times. In addition, the behavioral consequences of waiting time to be milked, the use of fetch pens, and the challenge of negotiating the robot entry point have not been examined. Such knowledge could inform better decisions on grouping strategies, group sizes, the timing of fetching and robot maintenance, along with the design characteristics of the robot entryway. Minimization of waiting time to be milked in an AMS should enhance cows' time available for rest and eat along with reducing the risk for lameness.

The objective of this study was to collect preliminary observational data from a single free-flow AMS herd to determine the milking time behavior for cows and identify potential factors responsible for variation in the time waiting to access the robot and be milked. We hypothesized a wide variability of waiting time at cow-level and that prolonged waiting time would negatively affect their lying time.

A commercial dairy farm in Wisconsin was recruited from an existing database of AMS farms that previously participated in a survey of management practices. The herd size was ∼180 Holstein lactating cows with no outdoor access and a mean daily milk yield of 40 kg/cow from 2.7 milkings/d. The farm, built in October 2016, had a free-flow traffic design (Lely Astronaut; Lely Industries NV) with 2 pens with mixed parities housed in a mechanical tunnel ventilated barn. One pen had 1 milking unit and 59 cows at the time of the study (this was the study pen) and the other pen had 2 milking units and 120 cows. In the study pen, the robotic milking unit was installed on the side of the barn, parallel to the pen. The fetch pen (a gated area used to gather cows that have not being milked within a predetermined interval) remained open throughout the day so cows could enter voluntarily but was gated closed when cows were fetched into the pen. When closed, cows could only exit through the robot. Pens had a 3-row stall layout with a single row of stalls against the sidewall and a double row of head-to-head stalls. All stalls were deep bedded with sand and there was a stocking density of one cow per stall. Pens had concrete flooring with alleys grooved parallel to the feedbunk and automatic alley scrapers. Feed was delivered 2 times/d, at 0600 and 0730 h. An automatic feed pusher ran every 1.5 h. Cows were fetched 3 times/d, approximately at 0500, 1300, and 1700 h.

Data were collected from July 9 to July 15, 2019. Producers' consent and a confidentiality agreement were received before data were collected. All methods were approved by the Institutional Animal Care and Use Committee (IACUC A005906) administered by the University of Wisconsin-Madison.

Behavior data were collected from one mixed-parity pen with one robotic milking unit. A purposive sample of 40 lactating cows was selected and balanced to reflect an equal proportion of primiparous and multiparous cows. The 40 cows were selected based on the order they entered the headlocks. This sample size was based on Ito et al. (2009), which determined that activity data on 30 cows for 3 d was representative of the herd for the evaluation of lying time. That same study also determined that a sampling period of 2 d provided estimates that were 90% accurate compared with a sampling period of 5 d. Cows with mastitis, surgeries, lameness, or close to dry off were excluded from the selection process. Data on parity, DIM, result of each visit to the milking robot (e.g., milking, refusal, failure), milking time and duration, and milk yield were automatically recorded and collected from the farm management AMS software.

Study cows were restrained in headlocks and their identification number was color marked with spray paint on the rump. This method of identification allowed for individual recognition and tracking on video when assessing individual cows' waiting behavior to access the robot. For 5 continuous days, 5 time-lapse cameras (TLC200Pro, Brinno, Brinno Inc.) were strategically placed in the study pen allowing for a complete view of the pen and study cows, ensuring that there were no blind spots. The cameras were mounted 4 to 5 m above the pen floor and were set to record continuously every 5 s at 10 frames per second. Lights over the pen were left on during the nights of the filming period to enable visibility for 24 h. During this period, no herd health management practices or scheduled maintenance to the milking robot were performed. From the 5-d period, 2 consecutive 24-h periods were selected to assess behavior data. This 2-d period of assessment was selected to mitigate factors hypothesized to affect cows' behavior, including the days when researchers were present, when stalls were bedded, and days with a higher environmental temperature.

During video analysis, a waiting area for milking was delimited to resemble a commitment or holding pen close to the milking unit. The boundaries of the waiting area were approximated using the gate of the fetch pen and the robot's exit gate. It included the area in front of the milking robot, which was a 4.5 m wide and a 7.5 m long alley, and the area in front of the fetch pen, which was 2.7 m wide and a 5.5 m long alley, for a total open area in front of the robot of approximately 49 m^2^.

Study cows were tracked continuously for the video recording period. For each study cow, the total duration of time spent in the waiting area and frequency of visits to the waiting area was recorded using Excel (Microsoft Corp.) from which daily waiting time, daily visit frequency, and visit duration were calculated for each cow. The time spent in the waiting area was determined as the time when each cow entered (i.e., start time; hh:mm:ss) and exited (i.e., end time; hh:mm:ss) the waiting area. Cows were considered to “exit” the waiting area if they accessed the robot (with a milking or nonmilking visit; also known as refusal) or left the approximated boundaries of the waiting area. Typically, it took <1 min for cows to pass through the alley in front of the milking unit. Based on this observation and on reports that cows walk at an average speed of 1.2 m/s (Flower et al., 2007), only observations in the waiting area that were equal to or longer than 1 min were considered. This was to ensure that observations of cows that entered the waiting area to use a stall or to pass through the alley were not considered as time spent waiting to be milked. Therefore, observations of cows that were within 1 min of entering the waiting area who went to use (e.g., perch, stand, or lie down) a stall in front of the waiting area were not considered. Observations of cows that exited the waiting area, but entered the waiting area again within 1 min, were considered part of the same waiting event. Observations of cows that entered the waiting area while other cows were being fetched or because of aggressive social interactions were not considered. Observations of cows that remained in the waiting area immediately after being milked were not considered. There were 482 observations generated on 40 cows throughout the 2-d observation period.

Lying behavior was recorded using activity data loggers (HOBO Pendant G Acceleration Data Loggers, Onset Computer Corp.) attached with bandaging wrap to each study cow's lateral lower right hind leg while the cow was restrained in headlocks. Data loggers were programmed to record at 30 s intervals as previously described (Ito et al., 2009; Solano et al., 2016). Lying bout duration (min/bout), lying bout frequency (bout/d), and total lying time (h/d) were computed using SAS software (version 9.4, SAS Institute Inc.) for each cow.

Indoor ambient temperature and relative humidity converted to the temperature-humidity index were recorded during the 5-d period as described by Nordlund et al. (2019).

All statistical analyses were performed using Stata 16 (StataCorp) considering the individual cow as the unit in all analyses. Descriptive summaries, relationships, and graphical data analyses were used to evaluate assumptions (i.e., independence, linearity of relationships, constant variance, normality of residuals) and screen all data (i.e., outliers, influential observations). Data for daily waiting time were square root transformed due to a positive skewed distribution.

The original data set of 482 observations (of repeated nature) were averaged per cow per day, resulting in a total of 80 observations. If cows entered the fetch pen, voluntarily or forced, ≥20% of the number of visits to the waiting area in 24 h, they were categorized as “fetch pen users” (e.g., if a cow visited the waiting area 10 times in 24 h and, of these, entered the fetch pen on ≥2 occasions, then she was categorized as “fetch pen user”). If cows used the fetch pen <20% of the number of visits in 24 h, they were categorized as “non-fetch pen users.” The value of 20% to define “fetch pen users” was selected after testing individually various cut-offs (i.e., 20, 25, 50, or 75% of the time in 24 h) as predictor variables in the models described below. Similarly, if cows were refused at the robot on ≥20% of the occasions they accessed it in 24 h, they were categorized as “frequent refused.” If cows were refused by the robot <20% of the occasions they accessed the robot in 24 h, they were categorized as “infrequent refused.” In general, cows may be refused by the robot if the predetermined milking interval is too short. The mean of daily lying time (h/d), bout frequency (bout/d), and bout duration (min/bout) were calculated for each cow.

Separate generalized linear mixed models for repeated measures were built for each outcome of interest. The outcomes of interest were daily waiting time to access the robot (min/d), average waiting time per visit to the waiting area (min/visit), number of visits to the waiting area (N visits/day), lying time (h/d), lying bout frequency (bout/d), and lying bout duration (min/bout). All models included cow as a random effect. Two-way interactions of interest were tested (e.g., parity and stage of lactation; parity and milk production). Multicollinearity, extreme values, overfitting, and confounding were examined by evaluation of estimates and graphical methods. Extreme values did not affect estimates; therefore, they were included in all analyses. Model comparison was based on assessment of adjusted R square, graphical evaluation of residuals, and Akaike information criterion.

This is the first study to explore the behavior of dairy cows waiting to access the robot to be milked in a single commercial free-flow AMS facility with a mixed-parity group of high milk production dairy cows. The results obtained are observational in nature and pertain to this specific situation. During the 2-d study period, the 40 study cows had complete and usable behavior and productivity data. Twelve cows were forced into the fetch pen at least once per day. The daily mean (± SD) milk yield for primiparous cows was 38.6 ± 9.1 kg and for multiparous cows was 49.4 ± 12.7 with an average milking frequency of 2.8 milkings per cow per day.

On average, cows visited the waiting area 6 ± 3 times/d, for 15 ± 20 min/visit, for a total daily waiting time to access the robot of 88 ± 60 min/cow (range 4.8 to 321.9 min; Table 1). The overall mean waiting time to access the robot of 1.5 h/d per cow compares favorably with the 2.7 h/d total time out of the pen waiting, milking and transferring to and from the milking parlor in parlor-milked herds (Gomez and Cook, 2010); however, the range among individual cows extended to over 5 h/d. Although some cows are remarkably efficient at being milked in an AMS, waiting less than 5 min/d, other cows seem to find robot access far more challenging. Visit duration to the waiting area was different (*P* < 0.05) depending on access to the milking unit and use of the fetch pen. Visit duration was longer if cows accessed the robot and were milked (18 ± 21 min/visit) and if cows used the fetch pen voluntarily (27 ± 31 min/visit) or were forced to use the fetch pen (30 ± 24 min/visit) compared with cows that did not access the robot or entered the fetch pen.

**Table 1:** Distribution of cow (n = 40) productivity, waiting, and lying time behavior variables in an automatic milking system with free-flow traffic design^1^

Variable	Mean ± SD	Median (IQR)	(Min–Max)	No. (%)
Daily milk yield (kg)	44 ± 12	42 (36–49)	20–88	—
DIM	132 ± 88	110 (58–209)	8–313	—
Parity	1.9 ± 1.2	1.5 (1–3)	1–6	—
1	—	—	—	20 (50)
2	—	—	—	9 (22)
3+	—	—	—	11 (28)
Waiting behavior				
Waiting time to access robot (min/d)	88 ± 60	77 (50–109)	5–322	—
Number of visits to waiting area (no./d)	6 ± 3	5 (3–7)	2–14	—
Visit duration in waiting area (min/visit)	15 ± 20	9 (4–19)	0.3–229	—
Accessed robot				
If accessed robot and milked	18 ± 21^a^	10 (4–24)	0.3–122	225 (47)^2^
If accessed robot and refused	15 ± 30^a^	7 (4–15)	0.2–229	69 (14)
If did not access robot	12 ± 12^b^	8 (4–15)	2–83	188 (39)
Fetch pen use				
If did not use the fetch pen	11 ± 12^a^	7 (4–14)	0.3–102	353 (73)^2^
If used the fetch pen voluntarily	27 ± 31^b^	18 (7–34)	1–229	102 (21)
If forced to use the fetch pen	30 ± 24^b^	23 (10–51)	0.3–80	27 (6)
Lying behavior				
Lying time (h/d)	10.7 ± 2.3	10.8 (9–12)	5–16	—
Bout frequency (n/d)	10.2 ± 3.7	10 (7–13)	3–20	—
Bout duration (min/bout)	70 ± 25	64 (52–83)	19–135	—

a,b Within a column and category, means without a common superscript differed (*P* < 0.05).

1 IQR = interquartile range; Min = minimum; Max = maximum.

2 Based on 482 observations generated on 40 cows throughout 48 h.

Distribution of the frequency of visits to the waiting area varied greatly throughout the day with periods of high and low activity at the robot. As was the case for an experimental AMS (Winter and Hillerton, 1995), cows in the current study had a crepuscular activity pattern with periods of high (i.e., 12 to 15 waiting visits within 1 h; around 0900, 1600, and 2200 h) and low (i.e., ≤5 waiting visits within 1 h; around 0200 and 1200 h) robot visit frequencies throughout the day. In general, as the number of waiting visits increased, the frequency of nonmilking visits increased. We inferred that management practices related to fetching, robot maintenance, and stocking rate of cows per robot need to consider a herd's specific periods of activity to reduce waiting time. Further research is needed on specific management practices that may have the largest impact on waiting time.

Daily waiting time to access the robot varied greatly among cows and was associated with DIM and parity (Table 2). It was longer for primiparous cows and decreased with increasing DIM, but this effect interacted with parity (Figure 1). Primiparous cows had longer waiting time in early lactation compared with multiparous cows. Toward late lactation, waiting time for primiparous cows was similar to the multiparous-cow pattern of waiting behavior, with fewer and shorter visits to the waiting area. “Fetch pen users” had longer daily waiting time compared with cows that rarely entered the fetch pen. It is possible that the long waiting times for primiparous cows in early lactation (mean = 2 h/d) in this study's mixed-parity group are because they must compete at the robot entryway with multiparous cows.

**Table 2:** Final generalized linear mixed models for 2 measures of daily waiting time to access the robot with cow-level factors in an automated milking system (AMS) with free-flow traffic design (n = 40 cows)^1^

Item	Square root of waiting time (min/d)		No. of visits to waiting area (no./d)	
	Estimate (95% CI)	*P*-value	Estimate (95% CI)	*P*-value
Intercept	10.74 (8.78 to 12.69)	<0.001	8.97 (8.47.262 to 10.67)	<0.001
Parity				
1	Referent		Referent	
2+	−3.39 (−6.03 to −0.75)	0.012	−3.57 (−5.73 to −1.40)	0.001
DIM	−0.02 (−0.03 to −0.003)	0.013	−0.03 (−0.04 to −0.01)	<0.001
Parity × DIM				
1 × DIM	Referent		Referent	
2+ × DIM	0.02 (0.00 to 0.04)	0.039	0.03 (0.01 to 0.04)	<0.001
Fetch pen users^2^		—		
No	Referent		Referent	
Yes	1.29 (−0.001 to 2.59)	0.050	−1.27 (−2.34 to −0.20)	0.020
Frequently refused at AMS^3^	—	—		
No	—	—	Referent	
Yes	—	—	1.99 (0.93 to 3.04)	<0.001

1 Waiting behavior and AMS data collected from 2 continuous 24-h periods.

2 Cows that used the fetch pen, voluntarily or forced, ≥20% of the time in 24 h.

3 Cows refused at the AMS ≥20% of the time in 24 h.

**Figure 1:**
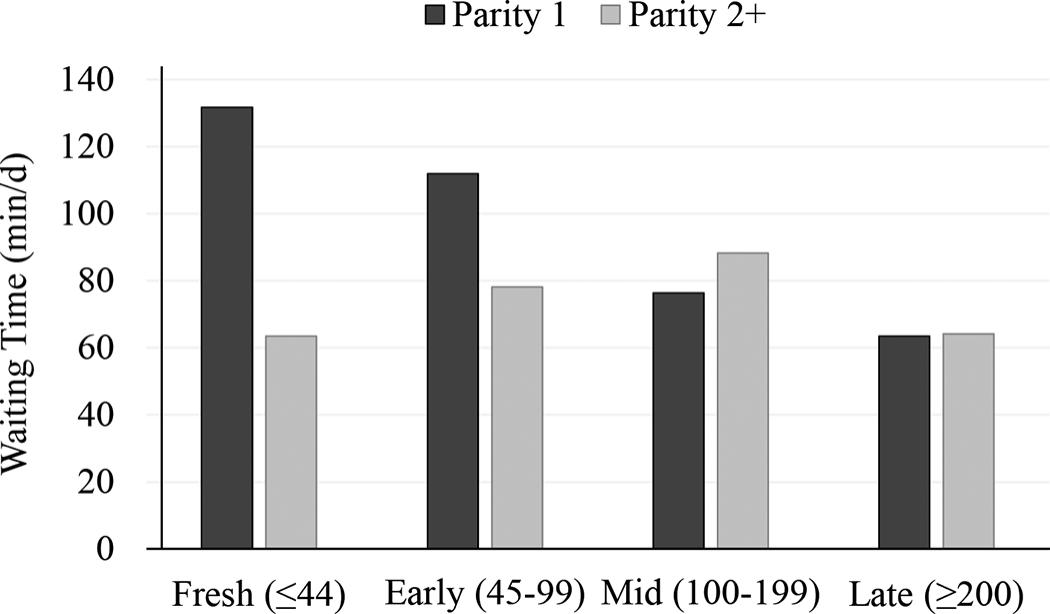
Daily waiting time (min) per parity and stage of lactation.

Time spent waiting to access the robot could be affected by other factors that influence the level of competition to access the robot and the design of the entry and exit area. A finding of this study that can explain the effect of the robot area entry design on waiting time was the observed ability for some cows to constantly exert influence over cows waiting in the fetch pen. They did this by forcing their way past the end of the swing gate, giving them priority for robot access over cows waiting in the fetch pen. This design feature could be modified by adding gating to protect the neck and shoulder of the cow waiting directly in front of the robot access gate, to ensure that dominant cows could not displace a subordinate by limiting their interaction to the rear of the waiting cow. In addition, primiparous cows of the study herd did not receive training to use the robot before entering the lactating group, in contrast to what has been recommended (Pitkäranta et al., 2019). These findings support the separate grouping of primiparous cows and multiparous cows along with training of nulliparous heifers to lessen this dominance effect, which appears to be particularly impactful early in lactation when primiparous cows are learning to access the robot.

Another finding which can help explain the impact of the robot area exit design was the observed agonistic behavior of some cows exiting the robot (after a successful milking) and displacing cows waiting in line to gain access to the robot. This displacement behavior appeared to be exacerbated during periods of high robot visit frequency. An AMS layout where cows exit away from the robot entrance (Pitkäranta et al., 2019) and are not able to interfere with cows waiting to access the robot could prevent this behavior.

Parity, DIM, and their interaction had a similar effect on daily number of visits to the waiting area compared with results of daily waiting time (Table 2). In contrast to multiparous cows, primiparous cows had a higher and wider range of mean daily number of visits to the waiting area. These decreased with increasing DIM, with more frequent (10 ± 2.3) visits in early lactation, decreasing to fewer (4 ± 2.2) in late lactation. Multiparous cows had 4.5 ± 1.3 visits/d in early lactation and 5.4 ± 3.1 in mid to late lactation. The very high visit frequency of primiparous cows in early lactation is perhaps indicative of their struggles for robot access. “Fetch pen users” had 1.3 fewer visits to the waiting area compared with cows that rarely entered the fetch pen. In contrast, “frequent refused” cows had an average of 2 additional visits to the waiting area compared with cows that were not refused.

Primiparous cows had a mean lying bout duration of 55.7 ± 19.2 min, with 12 ± 3.5 bouts daily for a mean total daily lying time of 10.4 ± 2.2 h/d. Multiparous cows had a mean lying bout duration of 84.3 ± 21 min, with 8.4 ± 2.9 bouts daily for a mean total daily lying time of 11.1 ± 2.3 h/d. Daily lying time decreased with increasing daily waiting time to be milked (*P* < 0.01). Daily lying time differed between cows with long (≥2 h) and short (<2 h) daily waiting times to access the robot. Cows with long daily waiting times had shorter daily lying time compared with cows with short daily waiting times (mean 9.5 vs. 11.1 h/d; *P* < 0.01), whereas daily bout frequency remained constant.

Similar to findings from conventional herds, cows with longer waiting times to access the robot had shorter daily lying times (Gomez and Cook, 2010; Charlton et al., 2014). The wide variation in waiting times observed within the herd could partly explain the small difference observed in herd mean lying times between conventional and robot milked dairy herds. Additionally, the short lying times observed may contribute to the high prevalence of lameness observed in AMS herds (King et al., 2016; Westin et al., 2016; Salfer et al., 2018) because in parlor-milked herds, longer milking times have been associated with a higher prevalence of lameness (Espejo and Endres, 2007). Factors that could reduce waiting times to be milked should be explored in lameness prevention strategies for AMS units.

The accurate and detailed analysis that this video-data capture method allowed provides an invaluable perspective into field-based factors that may affect cows' waiting behavior in a specific commercial facility design commonly used in North America. However, the observational nature of this study and the clustered data with the small sample size limited our ability to make strong inferences on how individual design features of the robot area influenced cows' behavior. Although the generalizability of our results is limited, this preliminary study informs our understanding of the impact of management practices and AMS facility design on cows' waiting behavior and provides a methodology by which more data can be accumulated across multiple AMS herds, both with free-flow and guided-flow systems.
